# Reply to: “Results from a biodiversity experiment fail to represent economic performance of semi-natural grasslands”

**DOI:** 10.1038/s41467-021-22310-0

**Published:** 2021-04-09

**Authors:** Sergei Schaub, Robert Finger, Florian Leiber, Stefan Probst, Michael Kreuzer, Alexandra Weigelt, Nina Buchmann, Michael Scherer-Lorenzen

**Affiliations:** 1 ETH Zürich, Agricultural Economics and Policy Group, Zurich, Switzerland; 2 ETH Zürich, Institute of Agricultural Sciences, Zurich, Switzerland; 3 Research Institute of Organic Agriculture (FiBL), Department of Livestock Sciences, Frick, Switzerland; 4 Bern University of Applied Sciences, School of Agricultural, Forest and Food Sciences, Zollikofen, Switzerland; 5 Leipzig University, Institute of Biology, Leipzig, Germany; 6 German Centre for Integrative Biodiversity Research (iDiv), Halle-Jena-Leipzig, Leipzig, Germany; 7 University of Freiburg, Faculty of Biology, Geobotany, Freiburg, Germany

**Keywords:** Biodiversity, Biodiversity, Ecosystem services

**Replying to** Tonn et al. *Nature Communications* 10.1038/s41467-021-22309-7 (2021)

In Schaub et al.^1^, we analyzed plant diversity effects on biomass yield, forage quality, quality-adjusted yield (biomass yield × forage quality) and revenues across different management intensities (extensive to very intensive) within the Jena Experiment (a large-scale grassland biodiversity experiment). For forage quality, we focused especially on metabolizable energy content and milk-production potential, variables rarely assessed economically in a biodiversity context. Our analysis suggested that plant diversity can substantially add to the milk-production potential yield (per unit of area) in semi-natural grasslands. This creates additional revenues from milk production. Our results showed that these plant diversity benefits can be as high as those from increasing management intensities within semi-natural grassland settings. In a recent comment, Tonn et al.^2^ challenged our findings, questioned their applicability for real-life systems and our calculation of the milk-production potential. We argue that their calculation offers a perspective on livestock performance, complementing our perspective of marginal benefits of plant diversity, and it shows that our main results for semi-natural grasslands are robust to differences in assessing milk-production potential yield.

Tonn et al.^2^ questioned if our results could be applied to real-life systems and they compared the experiment to resown, temporary, or permanent grasslands in different parts of their comment. However, the species used in our grassland plots belong to those of Central European *Arrhenatherion* semi-natural grasslands^3^, thus, clearly representing permanent grasslands. Using—among others—the same experimental data, Jochum et al.^4^ showed that plant diversity-ecosystem functioning relationships of biodiversity experiments (including the Jena Experiment) are realistic and that the plant communities in these experiments cover almost all of the variance observed in “real-world” communities. Jochum et al.^4^ further found that results from biodiversity experiments are largely insensitive to the exclusion of unrealistic communities, i.e., to communities that are not observed in the “real-world”. Moreover, it is worthwhile to highlight that plant diversity also increased biomass yield in another sub-experiment within the Jena Experiment, considering only highly performing species^5^. Similar to our analysis, various other studies showed no (or only small) plant diversity effects on forage quality in semi-natural and intensively managed grasslands (refs. ^6–9^). These findings highlight the relevance of our results in different contexts, including semi-natural grasslands. Moreover, we agree that different properties of grasslands can be considered, e.g., transgressive overyielding (i.e., that mixtures perform at least as good as the highest yielding monoculture^10^, best-performer approach^9^ or optimizing the mixture based on ecological production functions^11^. However, also considering these properties, positive economic benefits related to plant diversity have been shown for farmers in semi-natural grasslands and in intensively managed grasslands^9,11^. Consequently, we argue that our results are well within the range of real-life systems.

Tonn et al.^2^ also questioned whether the time span (2005–2007) was long enough for plant diversity to adapt to management changes. Findings from further studies of the same Management Experiment over a longer time span (2006–2009) showed that the plant diversity effect on biomass yield remained similar and that plant diversity only slightly declined with increasing management intensity^12,13^. In addition, plant diversity effects on forage quality (i.e., crude protein concentration) were found to be similar after plants had more time to adapt (2009)^8^. Moreover, differences observed in biomass yield and forage quality are clearly a result of plant ecophysiology and competition which change according to environmental and management drivers. In addition, also other plant diversity studies in more intensively managed grasslands and with different levels of plant diversity showed clear benefits from plant diversity (refs. ^9,11^). Therefore, we believe that the time span of our experiment was sufficient to reveal diversity responses to management changes.

Tonn et al.^2^ claimed that the exclusion of subplots with very low biomass yield, due to poorly performing plants (especially in the more intensive management), led to an overestimation of the plant diversity effect on biomass yields and quality-adjusted yields. We agree that under high-intensity management other species mixtures might prevail, which we have not studied as our focus was on semi-natural grasslands and the related species pool. However, considerable positive plant diversity effects on biomass yield have also widely been found with agronomic species mixtures specifically adopted to high-intensity leys^14^. Moreover, it is correct that we excluded subplots with very low biomass yield, but these were mostly communities with one or two species as in semi-natural grasslands the more diverse communities are more productive. Therefore, the exclusion reduced the number of plots on the lower end of the diversity gradient, and if anything, this rather led to an underestimation of the plant diversity effects.

Furthermore, Tonn et al.^2^ questioned our calculation of the milk-production potential and proposed an alternative calculation, assuming no supplementary feeding and that plant diversity effects on metabolizable energy contents existed. However, before assuming this, it would have been important to consider in a first step if the empirical analysis confirmed such plant diversity effects or not (i.e., if the Null Hypothesis of plant diversity effects ≠ 0 holds). Only then, inference on the economic significance of these effects (i.e., monetary consequences of plant diversity) should have been done in a second step. Conducting the second step without the first, as done by Tonn et al.^2^, can lead to unsubstantiated conclusions. This is important as in our original analysis we found no plant diversity effect on metabolizable energy contents for four out of five management intensities (i.e., all except intensive).

In our analysis, we applied the Swiss feeding recommendations for ruminants^15^ and estimated the benefits of plant diversity on potential milk production per area. We focused on marginal benefits in terms of revenues per area in semi-natural grasslands, without making any assumption about the in- or exclusion of the potential use of supplementary feeds. In contrast, Tonn et al.^2^ focused on metabolizable energy available for milk production after subtracting metabolizable energy requirements for maintenance. In addition, they calculated negative effects on dry matter intake assuming negative plant diversity effects on metabolizable energy contents and, consequently, digestibility and rumen emptying, following also Jans et al.^15^. We acknowledge the effects of forage quality on dry matter intake, but mainly for grazing systems without any complementary feeding. However, the here considered feeding recommendations^15^, like those in other countries, are based on the additivity principle, meaning that every unit of ingested energy counts, independent if derived from the roughage or from supplementary feeding. Indeed, in the majority of dairy systems, also in Germany where the Jena Experiment is located, the harvest is usually used as a diet component in combination with feeds of different compositions and quality to generate a balanced diet^16^. Accordingly, the harvest provides a defined amount of metabolizable energy per area, which adds to cover maintenance requirements and milk yield. Hence, and because forage quality stayed mainly constant in our experiment, it is justified to calculate the milk-production potential of harvests based on nutrient and energy amounts from the swards. Besides, our original analysis focused on identifying these marginal benefits of plant diversity and not on evaluating the entire feeding system. In addition, roughage intake from semi-natural swards was not always found to decrease along a decreasing energy gradient even when no supplementation was provided^17,18^, indicating that Tonn et al.’s^2^ argument of roughage intake depression may not hold true across all systems. Therefore, our original calculation is indeed valid to inform about marginal plant diversity benefits on milk-production potential yield, thus, on revenues in semi-natural grasslands. Finally, despite the differences in calculations, the findings by Tonn et al.^2^, presented in Fig. 1, support the main message of our analysis: plant diversity increases milk-production potential and revenues per area. Therefore, we argue that our original paper provided indeed important, correct insights into the economic marginal benefit of plant diversity in a semi-natural grassland.

## Reporting summary

Further information on research design is available in the Nature Research Reporting Summary linked to this article.

## Supplementary information

Reporting Summary

## Data Availability

No new data were generated for this reply. Data collected for the original study by Schaub et al.^1^ are available in Schaub et al.^19^.
